# Explaining Multiclass Compound Activity Predictions Using Counterfactuals and Shapley Values

**DOI:** 10.3390/molecules28145601

**Published:** 2023-07-24

**Authors:** Alec Lamens, Jürgen Bajorath

**Affiliations:** Department of Life Science Informatics, B-IT, LIMES Program, Unit Chemical Biology and Medicinal Chemistry, Rheinische Friedrich-Wilhelms-Universität, Friedrich-Hirzebruch-Allee 5/6, D-53115 Bonn, Germany

**Keywords:** machine learning, multiclass activity prediction models, dual-target compounds, single-target compounds, explainable artificial intelligence, counterfactuals, SHAP values

## Abstract

Most machine learning (ML) models produce black box predictions that are difficult, if not impossible, to understand. In pharmaceutical research, black box predictions work against the acceptance of ML models for guiding experimental work. Hence, there is increasing interest in approaches for explainable ML, which is a part of explainable artificial intelligence (XAI), to better understand prediction outcomes. Herein, we have devised a test system for the rationalization of multiclass compound activity prediction models that combines two approaches from XAI for feature relevance or importance analysis, including counterfactuals (CFs) and Shapley additive explanations (SHAP). For compounds with different single- and dual-target activities, we identified small compound modifications that induce feature changes inverting class label predictions. In combination with feature mapping, CFs and SHAP value calculations provide chemically intuitive explanations for model decisions.

## 1. Introduction

Compound property predictions are a prime application of machine learning (ML) in pharmaceutical research [1,2,3]. Properties of particular interest in drug discovery include, among others, physico–chemical properties, such as solubility, in vivo characteristics of small molecules, such as metabolic stability or cellular membrane permeability, and biological activity, the most popular molecular property for predictive modeling. Traditionally, much attention has been paid to predicting specific biological activities of compounds [3]. To this end, a variety of ML models have been, and continue to be, developed. Many of these models are derived for compound classification. For activity prediction, different tasks can be defined. For example, one often attempts to distinguish specifically active from randomly collected compounds or differentiate between compounds that are active against different targets. To address such predictions tasks, classification models using different algorithms are derived. Furthermore, activity predictions might also aim to differentiate between compounds with single- and multi-target activity [4,5], which generally represents a more challenging prediction task than distinguishing active from inactive compounds. Achieving high accuracy in activity predictions is a primary goal of ML. However, most ML models have black box characters, meaning that their predictions cannot be understood by human reasoning [6]. This presents a problem in many drug discovery applications, which especially applies when models derived for activity prediction are used to propose novel active compounds prospectively that then need to be synthesized and tested. In such situations, investigators must inevitably rely on predictions they cannot rationalize, which typically causes a natural reluctance to integrate predictive modeling into interdisciplinary small-molecule discovery cycles. Consequently, the impact of ML in the practice of early-phase drug discovery has remained limited over the years, despite the widespread use of ML models for compound property predictions. Accordingly, one would also like to understand prediction outcomes at the molecular level of detail. Explaining model decisions is a critical issue for further increasing the acceptance of ML for experimental design. Moreover, the rationalization of model decisions also becomes highly relevant in cases where predictions fail, which is particularly relevant for method development and assessment. Hence, for both practical and methodological reasons, there is increasing interest in explainable ML in the context of activity predictions and beyond [6,7,8]. Explainable ML is a part of the explainable artificial intelligence (XAI) field. ML models rely on computational representations, and predictions are ultimately determined by representation features. Importantly, XAI approaches are applicable to assess and quantify feature importance, which is a central theme in XAI [8]. To this end, local feature attribution methods are widely applied, which derive simple approximation models in the feature space vicinity of a test instance to quantify feature importance. For example, when linear approximation models are used, coefficients of feature variables provide a measure of relative feature importance. Additive feature attribution methods express the probability of a prediction as the sum of feature contributions. Fitting coefficients to feature contributions also represents the basis of feature weighting methods used in ML.

For the quantification of feature importance, the Shapley value concept, originating from cooperative game theory, has become popular in pharmaceutical research and other scientific areas [9]. Shapley values quantify the contribution of players to the collaborative gain in the game. In the adaptation of the Shapley value concept for XAI, players correspond to representation features (for example, molecular descriptors), and the game corresponds to the prediction of an individual test instance (such as a compound) [10]. Since ML models are typically derived using large numbers of features, the systematic calculation of Shapley feature-importance values over all possible ordered player/feature coalitions becomes very computationally expensive or infeasible. Therefore, the explicit calculation of Shapley values is usually replaced by local linear approximation models in the feature space vicinity of each test instance, giving rise to the Shapley additive explanations (SHAP) approach [10], which has also been adapted for explaining compound activity predictions [11]. Thus, the SHAP adaptation of the Shapley value concept also belongs to the spectrum of local feature attribution methods. A detailed description of different algorithms that can be used to estimate Shapley values, including SHAP, has recently been provided [10], which the further interested reader is referred to. A hallmark of the SHAP formalism is its ability to quantify the importance of features that are present or absent in test instances, which sets SHAP apart from other feature weighting methods. The sum of all positive and negative feature contributions quantified by SHAP values, then, represents the probability of a given prediction.

In addition to local feature attribution methods, as discussed above, a distinct approach adapted for XAI is the concept of counterfactuals (CFs) [12,13] that has also been applied in compound activity predictions [13]. CFs are defined as test instances with minimal feature changes leading to different class label predictions [1,14]. Thus, for a given test instance, one needs to identify other instances with minimal modifications and associated feature changes yielding a different prediction. For example, for active test compounds, a CF is a close structural analogue that is predicted to be inactive. Hence, CFs can be identified by database searching for existing compounds or generated computationally through analogue enumeration, followed by ML prediction. Different from SHAP calculations, there are no specific algorithmic requirements for CF identification. Importantly, despite their distinct nature, both the SHAP and CF approaches can be used in a model-agnostic manner, that is, applied to any ML model, regardless of its complexity, which represents an important criterion for XAI. However, SHAP and CF analyses have different specific aims. SHAP quantifies feature importance to predictions, while CFs aim to implicitly capture minimal feature changes in test instances converting class label predictions. Accordingly, features that are most critical for prediction outcomes, including opposing predictions for CFs, can be identified using SHAP, but CF analysis cannot replace the assessment of feature attribution. Therefore, we have reasoned that the SHAP and CF concepts are complementary, leading to the investigation of the combined SHAP–CF framework for XAI.

In this work, we have addressed an advanced compound activity prediction task from an XAI perspective. Instead of building binary classification models, multiclass models were derived to predict compounds having four different class labels. Specifically, we have attempted to differentiate between compounds with well-defined dual-target (DT) activity, corresponding single-target (ST) activity, and randomly chosen compounds (assumed to be inactive). These predictions then provided the basis for the core of our study: The SHAP and CF analysis were combined to rationalize predictions of the multiclass models. As introduced herein, a SHAP–CF analysis provides a new framework for explaining compound property predictions. Complemented by feature mapping on test compounds and visualization, chemically intuitive explanations of individual predictions were obtained. SHAP–CF analysis is shown to uncover structural features that preferentially occur in compounds with a given class label and to determine predictions. As such, the approach can be considered for a variety of applications in molecular ML and is also applicable to aid in compound design.

## 2. Results and Discussion

### 2.1. Study Design

**Figure 1:**
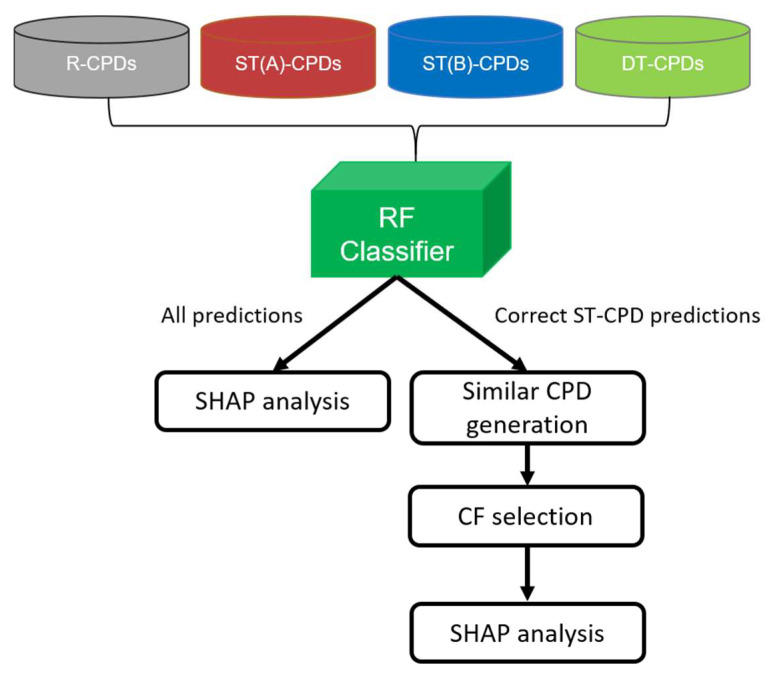
Combined SHAP and CF analyses of multiclass model predictions. The diagram summarizes the different steps of our analysis of DT- versus ST(A)/ST(B)- and R-CPD predictions using SHAP analysis and CFs.

The major goal of our study was to combine SHAP and CF analyses to rationalize ML model decisions for advanced compound activity predictions. Specifically, we aimed to explain predictions distinguishing compounds with activity against two different targets (dual-target compounds, DT-CPDs) from corresponding compounds with activity against the individual targets (single-target compounds, ST-CPDs) and randomly selected compounds (random compounds, R-CPDs). Therefore, we assembled a compound test system comprising two target pairs, for which a sufficient number of ST- and DT-CPDs were available for ML (see Section 3). For each target pair (A,B), a random forest (RF) multiclass model was generated to differentiate between four class labels, including DT-CPDs, ST-CPDs for target A (ST(A)-CPDs), ST-CPDs for target B (ST(B)-CPDs), and R-CPDs, which is considered to be inactive. The choice of the RF algorithm was motivated by earlier observations that predictions of binary RF classification models were often determined by features present in DT- but absent in corresponding ST-CPDs [5]. This prediction phenotype, with well-defined contributions of present and absent features for different class labels, provided a sound basis for CF analysis. Therefore, going beyond binary classification, we explored activity predictions at a higher level of complexity using multiclass models. Accordingly, for each target pair, we carried out systematic predictions using an RF multiclass model and conducted SHAP analysis to explain model decisions for four class labels. Then, for each correctly predicted ST(A)- and ST(B)-CPD, we searched for CFs (see Section 3) that were predicted to be DT-CPDs. Hence, for the identification of CFs, ST(A)- and ST(B)-CPDs served as the “base compounds” (Base-CPDs). Finally, SHAP analysis was repeated to understand why the class label predictions of Base-CPDs and corresponding CFs differed. Figure 1 illustrates the workflow of the combined SHAP and CF analyses for pairs of functionally distinct targets, including the 5-hydroxytryptamine receptor 1A and sodium-dependent noradrenaline transporter (target pair 1, TP1) and acetylcholinesterase and beta-secretase 1 (target pair 2, TP2). Notably, DT-CPDs of functionally unrelated targets are of particular interest in medicinal chemistry.

### 2.2. Multiclass Model Performance

**Figure 2:**
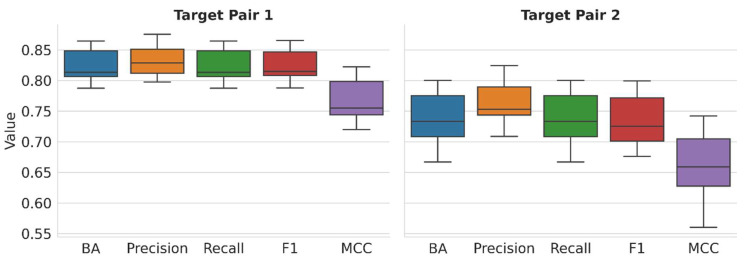
Prediction accuracy. Boxplots report the distribution of different performance metrics over 10 independent prediction trials on test set predictions for TP1 (**left**) and TP2 (**right**).

Figure 2 reports the performance of multiclass models on test set predictions over 10 independent trials and all class labels on the basis of different metrics (see Section 3).

For TP1, the median BA, precision, recall, and F1 values are consistently greater than 0.8, representing predictions with an accuracy of more than 80%. MCC ranges from −1 to +1, with a value of −1 accounting for consistently incorrect predictions, 0 for random, and +1 for consistently accurate predictions. For TP1, the observed median MCC value above 0.75 further reflects highly accurate predictions. For TP2, the prediction accuracy is lower than observed for TP1 but still satisfactory, especially for multiclass models, with median values of ~0.72–0.75 for the different performance measures and a median MCC value > 0.65. These multiclass predictions at different levels of accuracy provide an attractive basis for model explanation via XAI.

### 2.3. Rationalizing Model Decisions

**Figure 3:**
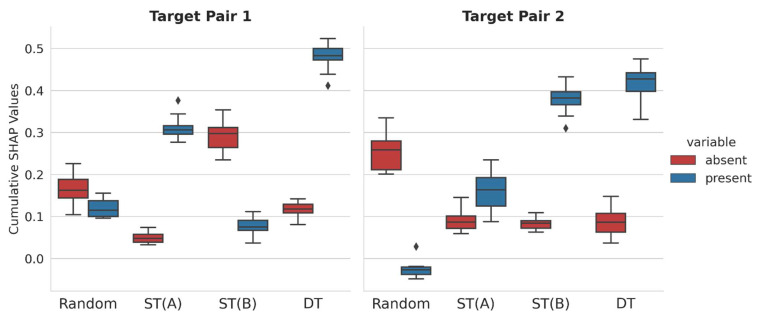
Cumulative SHAP analysis. Boxplots report the distribution of cumulative SHAP values for all correctly predicted test compounds belonging to four different classes over 10 independent prediction trials for TP1 (**left**) and TP2 (**right**).

For all correctly predicted test compounds, SHAP values of present and absent representation features (see Section 3) were calculated, summed over all test instances for each class label, and averaged for each prediction trial, yielding cumulative SHAP values for all class label predictions. By design, the calculation of cumulative SHAP values further extends the SHAP analysis of individual predictions across entire test sets. Figure 3 shows the distribution of cumulative SHAP values over 10 independent trials.

Positive cumulative SHAP values supported correct class label predictions, whereas negative values supported incorrect predictions. Furthermore, narrow value distributions across independent prediction trials indicated overall stable model explanations for test sets. Interestingly, different feature contribution patterns were detected. For TP1, correct predictions of R-CPDs were determined by features that were absent and, to a lesser extent, present in these compounds. However, the corresponding cumulative SHAP values were relatively small, indicating marginal feature contribution to correct predictions. By contrast, for TP2, correct R-CPD predictions were mostly driven by features absent in these compounds, whereas present features slightly opposed correct predictions. Furthermore, for TP1, features present in ST(A)-CPDs and absent in ST(B)-CPDs were largely responsible for correct predictions. Thus, in these cases, opposite feature contributions were decisive for ST-CPDs with different activities, which was a surprising finding. Partly different observations were made for TP2. Here, features present in ST(B)-CPDs also determined their correct predictions for TP2, but the contributions of features present and absent in ST(A)-CPDs with small cumulative SHAP values were responsible for the correct predictions in this case. Thus, for TP1 and TP2, the predictions of R-, ST(A)-, and ST(B)-CPDs were determined by different relative feature contributions. By contrast, for both TP1 and TP2, correct predictions of DT-CPDs were mostly determined by features that were present in these compounds, especially for the highly accurate TP1 predictions, whereas absent features made only marginal contributions. Accordingly, for both TP1 and TP2, DT-CPDs were indicated to contain structural features that set them apart from corresponding ST- and R-CPDs, hence providing a clear rationale for the correct predictions of DT-CPDs for distinct target combinations. The presence of structural features in DT-CPDs distinguishing them from ST-CPDs also provided a plausible and chemically intuitive explanation for their binding characteristics. Taken together, the cumulative SHAP analysis revealed different relative feature contribution patterns for determining the predictions of multiclass RF models. To obtain further insights, the SHAP analysis was complemented by studying the CFs.

### 2.4. Counterfactuals and Feature Mapping

**Figure 4:**
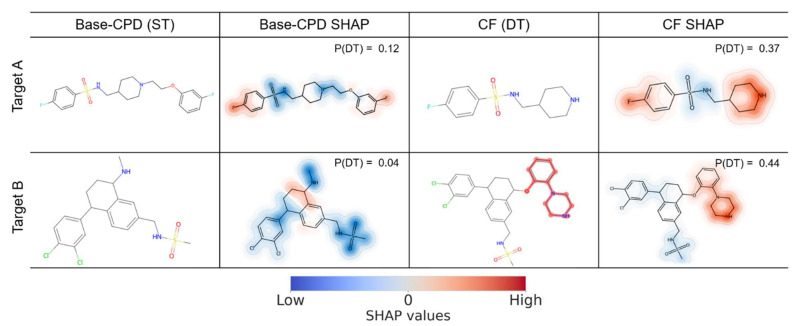
Mapping of present features determining counterfactual predictions for target pair 1. From left to right, the exemplary correctly predicted ST(A)- and ST(B)-CPDs from TP1 are shown, onto which present features contributing to a DT prediction based on SHAP values were mapped. Then, CFs are shown, with mapped present features determining the prediction of DT activity. The present features were mapped onto corresponding atoms, and atom-based feature values were summed and color-coded according to the spectrum shown at the bottom. Red and blue features support and oppose DT-CPD predictions, respectively. For each compound, P(DT) reports the probability of DT activity predicted by the multiclass model.

Given the consistent contributions of features that were present in DT-CPDs to correct predictions for different target pairs, we systematically searched for the CFs of ST-CPDs that were predicted to be DT-CPDs (see Section 3). Accordingly, these CFs represented a putative gain of function that should be associated with the occurrence of features preferentially found in DT-CPDs. For both target pairs, large numbers of qualifying CFs were obtained, with 3690 CFs for 139 Base-CPDs and 2346 CFs for 39 Base-CPDs for TP1 and TP2, respectively. For CFs, SHAP calculations were then repeated to identify the features that determine the predicted DT activity and compare them to the corresponding Base-CPDs with ST activity. Therefore, features decisive for the predictions were mapped on Base-CPDs and CFs. Figure 3 and Figure 4 show representative examples for TP1 and TP2, respectively. All representation features present in test compounds were mapped and color-coded according to their SHAP values. Features coloured in red supported the predictions of DT activity, whereas features coloured in blue opposed it. For each compound, the probability of DT activity produced by the multiclass model is reported. For multiple class labels, the highest-class label probability determined the prediction. Thus, for the CFs investigated herein, the probability of DT activity consistently represented the highest-class label probability.

In Figure 4, two exemplary ST-CPDs active against the targets of TP1 and the corresponding CFs are shown. For the Base-CPD active against target A (top), feature mapping revealed that the sulfonamide group strongly opposed the DT-CPD prediction together with a linker fragment connecting the aliphatic and aromatic rings, while the two fluorine-substituted benzene rings weakly supported the prediction of DT activity. These relative contributions resulted in a low probability of DT activity of 0.12, and the compound was correctly predicted as an ST(A)-CPD. In the corresponding CF, the linker fragment and attached fluorine-substituted benzene ring were removed, and the remaining piperidine ring strongly supported the DT-CPD prediction, which weakened the negative contribution of the adjacent sulfonamide moiety, leading to a probability of DT activity of 0.37.

In the Base-CPD active against target B (bottom), sulfonamide and methylamine moieties also strongly opposed the DT-CPD prediction together with a dichlorobenzene ring, leading to a very low probability of DT activity of 0.04. In the corresponding CF, the methylamino group was replaced by a 1-phenylpiperazine ring system, which strongly supported the prediction of DT activity, leading to a probability of 0.44. Substructure searches were performed to assess the significance of the highlighted moieties. It was confirmed that the piperidine and piperazine rings largely responsible for the predicted DT activity of CFs were preferentially found in actual DT-CPDs for TP1. Specifically, piperidine rings were present in 29% of DT-CPDs, while only 7% of ST-CPDs contained this substructure. The substructure searches also identified piperazine rings in 8% of DT-CPDs, but only in 1% of all ST-CPDs. Thus, the substitutions observed in the CFs of ST-CPDs mimicked the structural features of DT-CPDs, providing chemically meaningful explanations of predictions.

**Figure 5:**
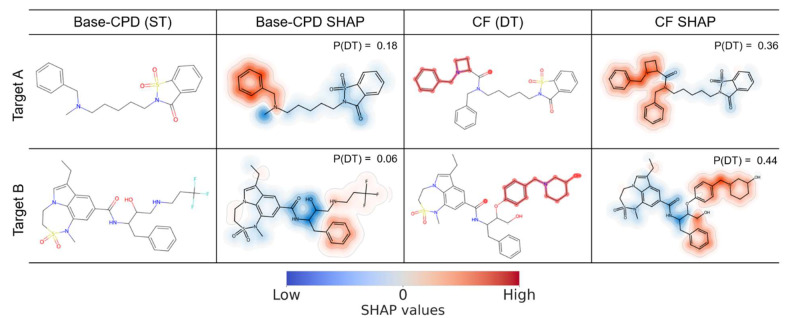
Mapping of present features determining counterfactual predictions for target pair 2. From left to right, exemplary correctly predicted ST(A)- and ST(B)-CPDs from TP2 are shown, onto which present features contributing to a DT prediction based on SHAP values were mapped. Then, CFs are shown with mapped present features determining the prediction of DT activity. Red and blue features support and oppose DT-CPD predictions, respectively. For each compound, the probability of DT activity predicted by the multiclass model is reported. The presentation is according to Figure 4.

Figure 5 shows two ST-CPDs active against the targets of TP2 and the corresponding CFs. In the Base-CPD active against target A (top), the benzylamine moiety strongly supported DT-CPD predictions, while the remaining substructure promoted the predictions of ST activity. In the corresponding CF, the addition of a phenylazetidine group strongly supported the prediction of DT activity, leading to a corresponding probability of 0.36.

In the Base-CPD active against target B, an amide bond and adjacent hydroxyl groups strongly opposed the DT-CPD prediction, while the tricyclic system on the left and the trifluorobutane and phenyl groups on the right made modest negative and positive contributions to the prediction of DT activity, respectively. The resulting probability of DT activity was very low (0.06), mostly due to the strong negative contributions in the center of the Base-CPD. In the corresponding CF, the substitution of the trifluorobutane group with a benzyl-piperidine moiety substantially increased the probability of DT activity to 0.44, thus inverting the prediction. Interestingly, a recent investigation attempted the design of dual inhibitors for TP2 [15]. The active compounds reported in this study contained a similar benzyl-piperidine substructure [15], consistent with the results of our analysis. Furthermore, substructure searches revealed that groups driving CF predictions were preferentially found in actual DT-CPDs. For example, the benzylamine moiety was detected in 64% of the DT-CPDs for TP2 but only in 27% of ST-CPDs. Moreover, the benzyl-piperidine moiety was contained in 48% of the DT-CPDs, yet only 10% of ST-CPDs. Hence, the CF analysis identified substructures implicated in DT activity and provided clear explanations of model decisions.

### 2.5. Concluding Discussion

ML models mostly produce black box predictions. While the inability to rationalize model decisions might be perceived as a general limitation of ML, especially if complex models are employed, consequences may vary depending on the scientific context and specific applications. For example, for hypothesis generation, black box predictions might be deemed acceptable; by contrast, for aiding in experimental design, they are often disregarded. Simply put, if experimentalists are challenged to plan their work on the basis of predictions that one cannot understand, they will often be reluctant, and understandably so. This situation directly relates to interdisciplinary research and the practice of drug discovery. In pharmaceutical research, ML already has a long history of more than 30 years; yet the impact of ML on experimental drug discovery programs remains limited to this day. With the increasing interest in ML in many different areas and the advent of highly complex models, there also is an increasing awareness of the intrinsic limitations of black box predictions, including the limited acceptance of such predictions to guide experimental work. Accordingly, there is growing interest in the methodologies from the XAI field to better understand model decisions and rationalize predictions. In medicinal chemistry, one of the premier applications of ML models of varying complexity is the prediction of compound properties, such as specific biological activities. Some explanation methods for predictions, such as feature weighting approaches, have already been employed for a long time to identify features that contribute most to given predictions. Moreover, new XAI concepts are being considered, including, among others, model-agnostic approaches for quantifying feature importance, such as the Shapley value concept and its SHAP adaptation for ML. Moreover, model decisions can also be dissected by determining the minimal feature sets that are responsible for the given predictions or investigating closely related test instances yielding opposing predictions, leading to the concept of CFs. While the SHAP analysis of compound property predictions has previously been reported and CFs have been investigated in the context of activity predictions in very few studies, to our knowledge, SHAP and CF analysis has, thus far, not been combined. In this work, we have attempted to do so in order to explain specialized activity predictions designed to distinguish compounds with DT activity from corresponding ST-CPDs. To further increase the complexity of the prediction task, multiclass models were derived to predict four class labels. The models produced overall accurate predictions that provided a sound basis for model explanation. The SHAP analysis revealed different relative contributions of features that were present or absent in DT-, ST(A)-, ST(B), and R-CPDs to accurate predictions. The ability to quantify the importance of absent features sets the SHAP approach apart from other feature-weighing approaches. For model explanation, this ability was of critical relevance, at least in this case. While the prediction characteristics differed for R-, ST(A)-, and ST(B)-CPDs, depending on the target pair, predictions of DT-CPDs were consistently driven by features present in these compounds. To better understand these predictions, the CFs were systematically generated and subjected to SHAP analysis. Feature mapping provided interpretable visualizations of prediction outcomes. The combined SHAP and CF analysis revealed moieties that were largely responsible for converting the prediction of ST activity into DT activity and led to the identification of substructures that were decisive for the predictions and preferentially occurred in DT-CPDs. Taken together, the results of our proof-of-concept investigation established the complementary nature of SHAP and CF analysis for rationalizing advanced compound activity predictions. Current limitations of the SHAP-CF approach for compound predictions include that SHAP is a local approximation method and that there is no guarantee that CFs can be found for given test instances. The latter limitation generally applies to CFs. However, for molecular class label predictions, the probability of identifying structural analogues of test compounds with predictions is high. This is the case because an abundance of structural analogues can be enumerated computationally for given compounds and immediately subjected to predictions aiming to identify CFs. The former limitation can principally be addressed by replacing SHAP calculations with exact Shapley values. For example, ongoing efforts in our laboratory focus on devising algorithms for the calculation of exact Shapley values for given ML methods, such as support vector machines. While such approaches are no longer model agnostic like SHAP, they can also be combined with CF analysis in the same way. The SHAP-CF approach should also be attractive for investigating other compound property predictions in drug discovery and can also be directly applied to rationalize predictions of activity cliffs that are formed by structural analogues with large differences in potency, representing another interesting opportunity for future research. Moreover, although the SHAP-CF approach was conceived in the context of compound activity predictions, this methodological framework is not confined to drug discovery applications but is also applicable to prediction tasks in many other areas.

## 3. Materials and Methods

### 3.1. Compounds and Target Pairs

Compounds were selected from CHEMBL (version 30) [16] on the basis of the following criteria. All compounds with activity comments, such as “inactive”, “not active”, “inconclusive”, or “potential transcription error” were initially discarded. Candidate compounds were required to have a molecular mass of less than 1000 Da and a potency value higher than 10 µM. Only compounds with a standard potency measurement (K_i_, K_d_ or IC_50_) and numerically specified potency value (standard relation: “=”) were retained. In addition, a direct target interaction at the highest level of confidence (target confidence score 9) was required. Finally, publicly accessible filters were applied to remove potential assay interference compounds [17,18,19].

Two pairs of functionally unrelated targets were selected for which sufficient numbers of qualifying DT- and corresponding ST-CPDs were available for ML (notably, unrelated targets with sufficient numbers of DT-CPDs are rare). The data sets for these two target pairs were balanced by undersampling compound majority classes (DT-CPDs). In addition, the corresponding numbers of R-CPDs meeting our selection criteria were randomly extracted from ChEMBL. For target pair 1 consisting of 5-hydoytryptamine receptor 1A and the sodium-dependent noradrenaline transporter, 98 DT-CPDs, ST(A)-CPDs, ST(B)-CPDs, and R-CPDs were obtained. For target pair 2 (acetylcholinesterase and beta-secretase 1), 50 DT-CPDs, ST(A)-CPDs, ST(B)-CPDs, and R-CPDs were obtained.

### 3.2. Machine Learning

Multiclass RF models [20] were derived using the *RandomForestClassifier* function of scikit-learn [21]. An implementation of the extended connectivity (atom environment) fingerprint with bond diameter 4 [22] comprising 4096 bits was generated using RDKit [18] and used as the molecular representation. Atom environment features are generated in a molecule-specific manner and yield bond diameter-dependent feature sets that typically vary in size depending on the compound. These feature sets are then encoded as a binary fingerprint of constant length such that features originally represented as integer strings are mapped to individual bits. By design, many atom environment features are overlapping yet distinct. The variable indicating the class label (DT, ST(A), ST(B), or Random) was numerically encoded.

#### 3.2.1. Training and Hyperparameter Optimization

For each target pair, models for 10 independent prediction trials were derived by randomly dividing the training and test data into partitions of 70% and 30%, respectively. Compounds were partitioned using the *StratisfiedShuffleSplit* function of scikit-learn [20]. For hyperparameter optimization, random training set partitions of 70% and 30% were generated. Hyperparameter optimization included the minimal number of samples for a leaf node (1, 2, 5, 10), the minimal number of leaves required for a split (2, 3, 5, 10), and the number of decision trees (25, 50, 100, 200, 400). For the remaining hyperparameters, default settings were used. Following hyperparameter optimization, the final prediction model was derived based on the entire training set.

#### 3.2.2. Performance Metrics

The ability of the multiclass models to correctly classify test compounds was determined using five performance metrics, including balanced accuracy (BA) [23], precision, recall, F1 score (F1) [24], and Matthew’s Correlation Coefficient (MCC) [25]:

$$\mathrm{BA}=\frac{1}{2}\left(\mathrm{TPR}+\mathrm{TNR}\right)$$

$$\mathrm{Precision}=\frac{\mathrm{TP}}{\mathrm{TP}+\mathrm{FP}}$$

$$\;\mathrm{Recall}=\frac{\mathrm{TP}}{\mathrm{TP}+\mathrm{FN}}$$

$$F1=2\times \frac{\mathrm{TP}}{2\mathrm{TP}+\mathrm{FP}+\mathrm{FN}}$$

$$\mathrm{MCC}=\frac{\mathrm{TP}\times \mathrm{TN}-\mathrm{FP}\times \mathrm{FN}}{\sqrt{\left(\mathrm{TP}+\mathrm{FP}\right)\left(\mathrm{TP}+\mathrm{FN}\right)\left(\mathrm{TN}+\mathrm{FP}\right)\left(\mathrm{TN}+\mathrm{FN}\right)}}$$

### 3.3. SHAP Analyis

SHAP values can be calculated in different ways, depending on the ML algorithms used [10,26]. For our analysis, SHAP values were calculated using the TreeExplainer algorithm with “interventional” feature perturbation [27]. For each model, the entire training data set served as the background sample. SHAP calculations yield importance values for individual features quantifying their contribution to a prediction. Because importance values are calculated for individual features, they are not affected by potential feature correlation (but might reveal correlated features that comparably contribute to a prediction).

### 3.4. Feature Mapping

Since atom environment features consist of atom sequences, they can be mapped back to test compounds by labeling all of the atoms a feature contains. For features with SHAP-derived importance values, this is elegantly facilitated by assigning the SHAP value of a feature to each participating atom. If an atom participates in multiple features, its final value is the sum of all assigned feature values. Then, colored coding is applied using a continuous color spectrum to visualize the atomic contributions to predictions. Thereby, atom-based feature mapping can identify coherent substructures resulting from overlapping atom environment features and visualize their contributions to predictions.

### 3.5. Counterfactuals

For each correctly predicted ST-CPD, CFs were generated using the EXMOL package [14]. Initially, narrow chemical space around a Base-CPD was populated with candidate compounds using the superfast traversal, optimization, novelty, exploration and discovery (STONED) method using the synspace option [28]. Then, the multiclass models were used to predict sampled CF candidate compounds. CFs represented compounds predicted to be DT-CPDs. For predictions producing CFs, SHAP values for comparison with Base-CPDs were calculated using TreeExplainer.

Herein, CFs were generated using frequently observed substituents in active compounds not specific to the prediction task. However, the way in which structural analogues are computationally generated in the search for CFs can be varied, as discussed above, and also adjusted to specific prediction tasks, if required.

## Data Availability

No new data were created.

## Sample Availability

The compound test system is available from the authors.
